# Pulmonary isolation and clinical relevance of nontuberculous mycobacteria during nationwide survey in Serbia, 2010-2015

**DOI:** 10.1371/journal.pone.0207751

**Published:** 2018-11-21

**Authors:** Ivana Dakić, Irena Arandjelović, Branislava Savić, Snežana Jovanović, Mirjana Tošić, Tatjana Kurucin, Dragana Vuković

**Affiliations:** 1 Institute of Microbiology and Immunology, Faculty of Medicine, University of Belgrade, Belgrade, Serbia; 2 Department of Microbiology, Clinical Center of Serbia, Belgrade, Serbia; 3 Department of Microbiology, Municipal Institute for Pulmonary Diseases and Tuberculosis, Belgrade, Serbia; 4 Center for Microbiology, Virology and Immunology, Institute for Pulmonary Diseases of Vojvodina, Sremska Kamenica, Serbia; National Institute of Infectious Diseases, JAPAN

## Abstract

The rates of pulmonary colonization and disease due to nontuberculous mycobacteria (NTM) appear to be increasing globally, but diversity of species recovered as well as clinical relevance of NTM isolates differ considerably by geographic region. The first nationwide study of isolation frequency and clinical significance of NTM in Serbia included all patients with respiratory specimens yielding a positive NTM culture over the six-year period, 2010–2015. We analyzed trends in annual NTM isolation and NTM pulmonary disease (PD) incidence rates, with NTM PD cases defined in accordance with microbiological criteria established by the American Thoracic Society/Infectious Diseases Society of America (ATS/IDSA). 777 pulmonary NTM isolates were collected from 565 patients, of whom 126 (22.3%) met the ATS/IDSA criteria. The annual NTM isolation and NTM PD incidence rates per 100,000 changed over 2010–2015 from 0.9 to 1.6 (p = 0.1746) and from 0.18 to 0.48 (p = f0.0040), respectively. Both isolation and disease rates increased considerably with age, while higher NTM PD rates were also associated with residence in urbanized areas. Diversity of NTM species isolated was shown to be region-specific, with *M*. *xenopi* as the most prevalent species (17.3%), and increasing isolation rates of rapid growing mycobacteria (RGM) (p = 0.0072). *M*. *xenopi* was also the most common cause of NTM PD (28.6%), followed by RGM (27.8%). With 73% clinically relevant isolates, *M*. *abscessus* was identified as the most clinically relevant NTM species. While NTM PD obviously remains a rare disease in Serbia, the overall results justify recognition of NTM as pathogens of rising importance, and require further characterization of their epidemiology in the country.

## Introduction

Nontuberculous mycobacteria (NTM), i.e. mycobacteria other than *Mycobacterium tuberculosis* complex (MTBC) and *M*. *leprae*, are natural inhabitants of different environments, such as soil, water, water aerosols, and dust [1]. Human exposure to NTM is, therefore, common and practically inevitable, but with a noticeable geographic variability in prevalence of different NTM species isolated from clinical samples [2–4]. Although pathogenicity of NTM for humans is generally estimated as low, numerous reports of increased rates of isolation of NTM from clinical samples demonstrate their potential as emerging opportunistic pathogens [5–8]. NTM are associated with a range of infections, with pulmonary disease (PD) being the most common.

An increase in the frequency of NTM detection in clinical samples as well as in the number of patients with NTM PD has in particular been noted in countries where the incidence of tuberculosis (TB) has declined [5,9]. The increase in NTM isolation has also been related to improvements in laboratory methodology, allowing better recovery and accurate identification of these bacteria [5,10]. Both of these factors that may contribute to the increased recognition of NTM as human pathogens are effective in Serbia. Although TB remains major mycobacterial disease in the country, the burden of the disease has been significantly reduced over the recent years. Namely, TB notification rate in 2003 was 37 while in 2015 was 12.7 [11]. The decrease may be mainly ascribed to resources provided for TB control by the Global Fund in 2004 and 2010. As far as laboratory diagnostics is concerned, molecular identification of all mycobacterial cultures isolated in the country was introduced in 2008, and fully implemented in 2010. The National reference laboratory (NRL) performs the molecular tests for all laboratories included in Serbian TB laboratory network, which is a functionally integrated system of 29 laboratories providing mycobacterial laboratory services for entire population. The centralized identification and collection of mycobacterial cultures as well as patients’ data afforded the opportunity to carry out the first comprehensive analysis of the scope and importance of NTM in the country.

In this retrospective laboratory-based study we analyzed trends in NTM pulmonary colonization and disease in Serbia over a six-year period. We also aimed to establish diversity and clinical relevance of NTM species recovered from pulmonary specimens, and to identify factors associated with NTM colonization and NTM PD.

## Materials and methods

### Data source

Data were collected retrospectively from the NRL database. All patients with pulmonary specimens yielding a positive NTM culture from January 1^st^ 2010 to December 31^st^ 2015 were included. We recorded patients’ demographic and microbiological data such as age, gender, region of residence, specimen collection date, type of specimen, source laboratory, and NTM species identified.

### Microbiological analyses

**Fig 1:**
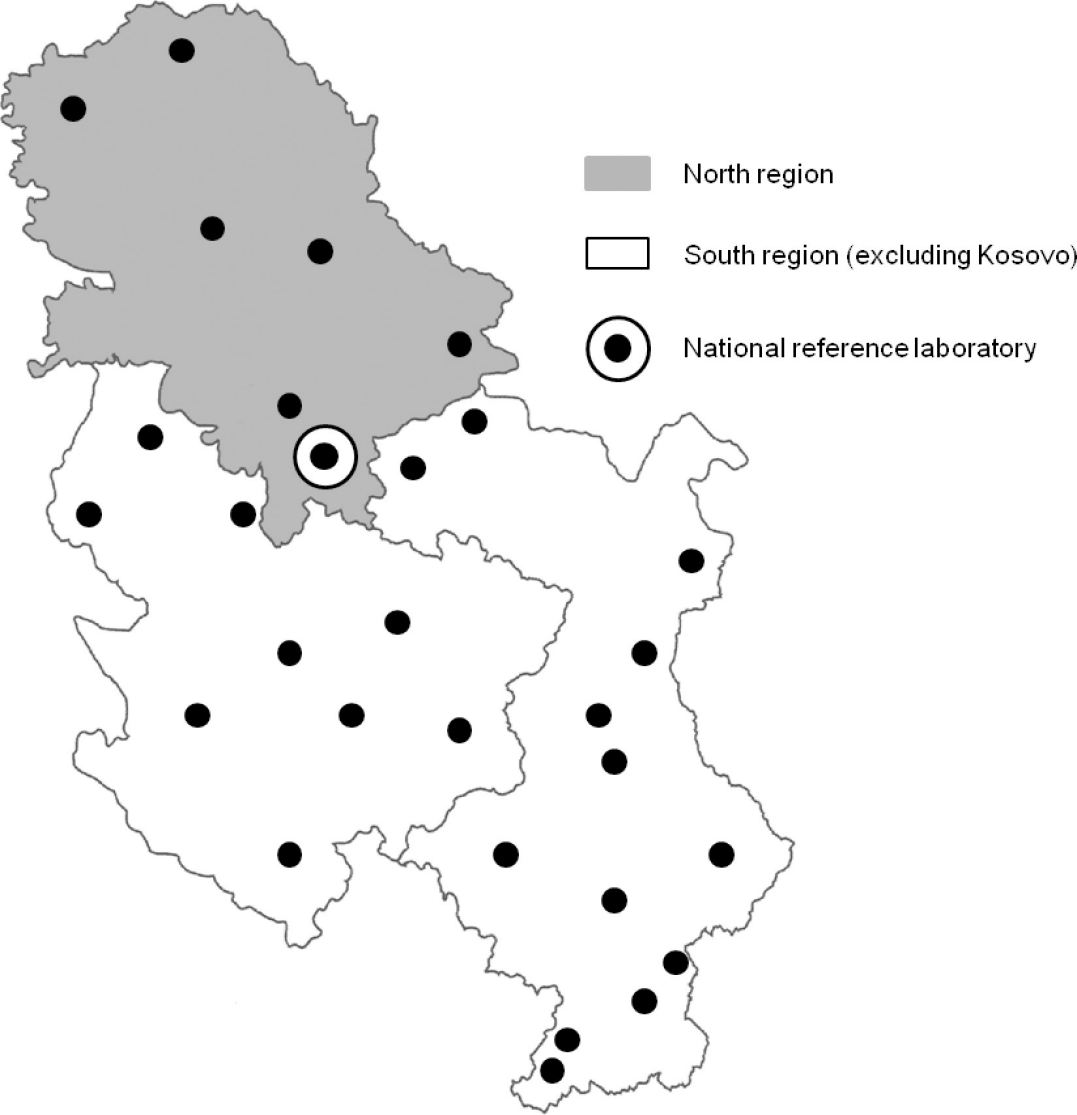
Tuberculosis laboratory network in Serbia (excluding Kosovo).

The national TB laboratory network (Fig 1) entitled to carry out mycobacterial laboratory diagnostics for all public healthcare facilities dealing with diagnosis, treatment, and follow-up of the patients with acute and chronic pulmonary disease (81 outpatient pulmonology departments, 29 general and special hospitals, four clinical centers, three military healthcare institutions, and one center providing healthcare in correctional facilities) participated in the study. The laboratories in the network performed preliminary identification of mycobacterial cultures based on their phenotypic traits, i.e. microscopical and cultural characteristics. The cultures were then sent to the NRL for molecular identification by line probe assays GenoType MTBC [12] and GenoType Mycobacterium CM [13] (Hain Lifescience, Nehren, Germany). All strains were tested by the GenoType MTBC assay which differentiates MTBC species, and the isolates not recognized as members of the complex were further tested by the GenoType CM. The version of the GenoType CM used during the study period (VER 1.0) enabled identification of 14 most relevant NTM species. In accordance with the manufacturer’s instructions, the isolates not identifiable to the species level but recognized as members of the genus *Mycobacterium* were designated *Mycobacterium* sp.

### Clinical significance of NTM

We used microbiological criteria established by the American Thoracic Society/Infectious Diseases Society of America (ATS/IDSA) to determine whether NTM were the likely cause of PD [5]. The pulmonary case had positive culture results from at least two separate expectorated sputum samples or positive culture result from at least one bronchial wash or lavage, while a noncase had a single positive sputum culture for a distinct NTM species. The level of NTM species clinical relevance was estimated as proposed by van Ingen [14]. For each NTM species the proportion of patients who met the ATS/IDSA criteria out of total number of patients per species was calculated.

### Data analysis

One isolate per patient was eligible for calculating NTM isolation frequency rates. For patients with multiple NTM isolations only the first isolate that occurred over the study period was taken into account, while for patients whose samples yielded different NTM species, each species was counted separately. Annual NTM isolation frequency rates were assessed as the number of patients with an NTM isolate recorded in a given year divided by the mid-year population and expressed per 100,000. The cases of NTM PD were considered incident in the year they met criteria for the previously described case definition, excluding patients whose specimens yielded *M*. *gordonae* as a typical contaminant. The annual incidence rates of NTM PD were calculated as the number of new cases in a given year divided by the mid-year population and expressed per 100,000. Bearing in mind chronic nature of NTM PD and possible misclassification of previously existing cases as incident, we also calculated prevalence for the periods 2011–2012 and 2014–2015 by taking into account total numbers of cases existent over the periods [8,15]. Average annual NTM isolation and NTM PD rates during the study period were evaluated for the following demographic factors: gender, age, and region of residence. Data on the overall and gender-, age-, and region-specific population of Serbia during the period were provided by the Statistical Office of the Republic of Serbia (http://webrzs.stat.gov.rs). Age groups were defined as 0–19 years, 20–39 years, 40–59 years, and 60 or more years. The country is divided into two geographically distinct regions, the North including Belgrade as the capital and largest city of Serbia, and the mostly rural South region. Gender-, age-, and region-specific isolation frequency and NTM PD rates were compared by the chi-square test, while linear regression was used for analyses of temporal trends in the rates. A p value of 0.05 or less was considered statistically significant. The data were analyzed with GraphPad Prism 6 software (GraphPad Prism Software Inc., La Jolla, USA).

The study followed the principles of Declaration of Helsinki, and guidelines provided by the Ethical Committee, Faculty of Medicine, University of Belgrade, which approved the study protocol (Decision Number 2650/VI-24). The Ethical Committee waived the requirement for informed consent, since this was a retrospective and noninterventional study which analyzed the data collected from routine laboratory practice. Patients’ data were anonymized by authorized laboratory personnel prior to analysis.

## Results

### NTM isolation and infection rates

During the six-year study period 777 pulmonary NTM isolates were collected from 565 patients. Men accounted for 51.7%, and the age of the patients ranged from 2 to 89 years, with average of 63 years. The annual isolation frequency rates of NTM (Table 1) increased from 0.9/100,000 to 1.6/100,000, with average annual rate of 1.3/100,000, but the trend across time was not significant. The average annual NTM isolation rates did not differ between men and women as well as between the two regions, while a marked increase was noted with increasing age category. With 3.38 per 100,000 the average annual frequency was by far the highest in 60 years and over age group. Analysis of gender-specific rates revealed an increasing trend in colonization rates in male individuals over time (p = 0.017) (S1 Table), while the trend for women remained unchanged. Men-to-women ratio differed only in the oldest age group, with men being more frequently colonized (S2 Table).

**Table 1:** Nontuberculous mycobacteria (NTM) isolation frequency rates and nontuberculous mycobacterial pulmonary disease (NTM PD) incidence rates stratified by year, gender, age, and region, Serbia, 2010–2015.

	Baseline population	NTM isolation frequency		p value	NTM PD incidence^b^		p value
		n	Rate^a^ (95% CI)		n	Rate^a^ (95% CI)	
All cases	7184110	565	1.30 (1.19–1.41)		126	0.29 (0.24–0.34)	
**Year**							
2010	7236519	65	0.89 (0.68–1.12)	0.1746	12	0.18 (0.07–0.26)	0.0040
2011	7236519	114	1.57 (1.28–1.86)		17	0.23 (0.12–0.35)	
2012	7201497	78	1.08 (0.84–1.32)		21	0.30 (0.17–0.42)	
2013	7166553	86	1.20 (0.95–1.45)		19	0.27 (0.15–0.38)	
2014	7131787	106	1.48 (1.19–1.78)		28	0.43 (0.25–0.54)	
2015	7131787	116	1.63 (1.33–1.92)		29	0.48 (0.26–0.55)	
**Gender**							
Male	3498324	292	1.39 (1.23–1.55)	0.4240	66	0.31 (0.24–0.39)	0.9130
Female	3685786	273	1.23 (1.09–1.38)		60	0.27 (0.20–0.34)	
**Age (years)**							
0–19	1425736	11	0.13 (0.05–0.20)	0.000	2	0.02 (0.0–0.06)	0.000
20–39	1905348	40	0.35 (0.24–0.46)		10	0.09 (0.03–0.14)	
40–59	2036628	146	1.19 (1.00–1.39)		37	0.30 (0.21–0.40)	
≥60	1816398	368	3.38 (3.03–3.72)		77	0.71 (0.55–0.86)	
**Region**							
North	3584005	299	1.39 (1.23–1.55)	0.1650	101	0.47 (0.38–0.56)	0.000
South	3600105	266	1.23 (1.08–1.38)		25	0.12 (0.07–0.16)	

^a^per 100,000 population

^b^excluding *M*. *gordonae*.

Of the 565 patients with NTM isolates, 126 (22.3%) met the microbiological ATS/IDSA case definition criteria. An average age of the patients was 65.4 years (range 2–88), and men accounted for 52.4% of all cases. The number of cases identified from a bronchial wash or lavage specimen was 22 (17.5%), while the remaining cases were identified according to the multiple positive sputum samples. The maximal number of positive sputum specimens per patient was 12, but majority of the patients, i.e. 63 and 27 had two and three, respectively.

Annual incidence rates of NTM PD (Table 1) increased considerably from 0.18 in 2010 to 0.48 in 2015. The average annual incidence rate was 0.29/100,000 population. Average annual NTM PD incidence during 2010 to 2015 increased significantly with age, and was considerably higher in the North region (Table 1). Similarly to the isolation frequency rates, an increasing trend in NTM PD incidence was observed in men throughout the study period (S1 Table). Age- and sex-stratification of NTM PD cases revealed similar proportions of male and female patients in all age groups apart from the >60 years group (S2 Table). Among these patients the disease was more common among men than women with average annual incidence rates of 0.96/100,000 and 0.51/100,000, respectively (p = 0.0392). Comparison of annualized NTM PD prevalence for 2011–2012 and 2014–2015 periods showed a considerable increase from 0.31 cases per 100,000 (95% CI, 0.22–0.40) to 0.47 cases per 100,000 (95% CI, 0.36–0.58), respectively (p = 0.0073).

**Fig 2:**
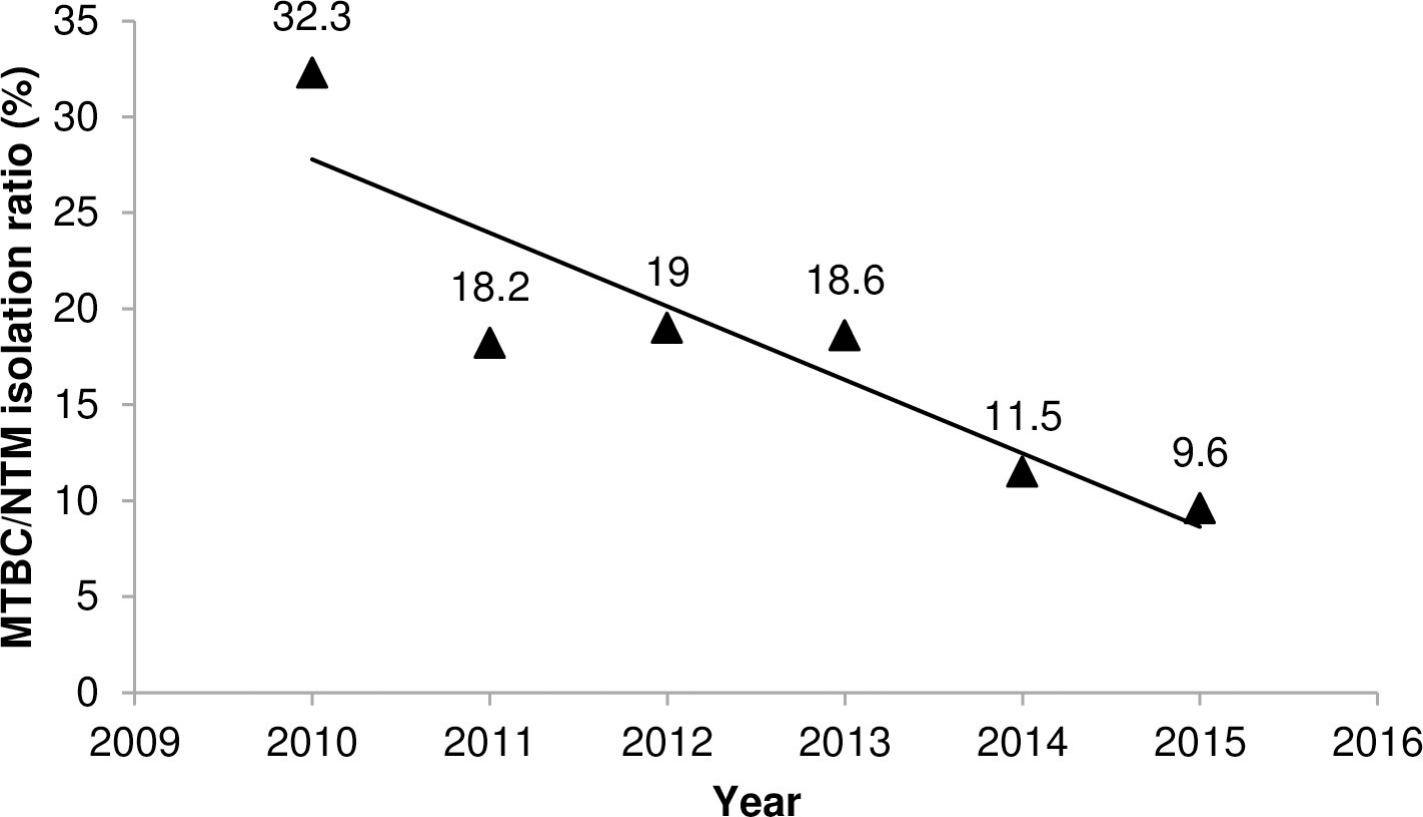
Ratio of *M*. *tuberculosis* complex (MTBC) and nontuberculous mycobacteria (NTM) pulmonary isolations in Serbia, 2010–2015.

In order to better understand the perceived increase in NTM isolation and infection, we reviewed data on all pulmonary isolates submitted for mycobacterial culture in Serbia between 2010 and 2015. Consistent with a marked decline in TB incidence, the annual number of respiratory specimens analyzed significantly decreased throughout the study period (Table 2), as well as the ratio between the annual fractions of MTBC isolation and the fractions of NTM isolation from pulmonary specimens depicted in Fig 2 (p = 0.0152). However, the fractions of positive specimens remained stable, with clearly increasing trend in the annual fractions of respiratory samples that were positive for NTM (p = 0.0068).

**Table 2:** Overview of mycobacterial analyses of pulmonary specimens in Serbia, 2010–2015.

Year	Pulmonary specimens		Culture positive specimens for mycobacteria	
	Submitted, n	Annual change, %	All, n (%)	NTM, n (%)*
2010	90720	-	2674 (2.9)	80 (3.0)
2011	85621	-5.6	2598 (3.0)	136 (5.2)
2012	78380	-8.5	2235 (2.8)	111 (5.0)
2013	76069	-2.9	2197 (2.8)	113 (5.1)
2014	76554	0.6	1916 (2.5)	154 (8.0)
2015	75890	-0.8	1954 (2.6)	183 (9.4)

*The proportions calculated from total number of culture positive specimens for mycobacteria.

### Diversity and clinical relevance of NTM species

**Fig 3:**
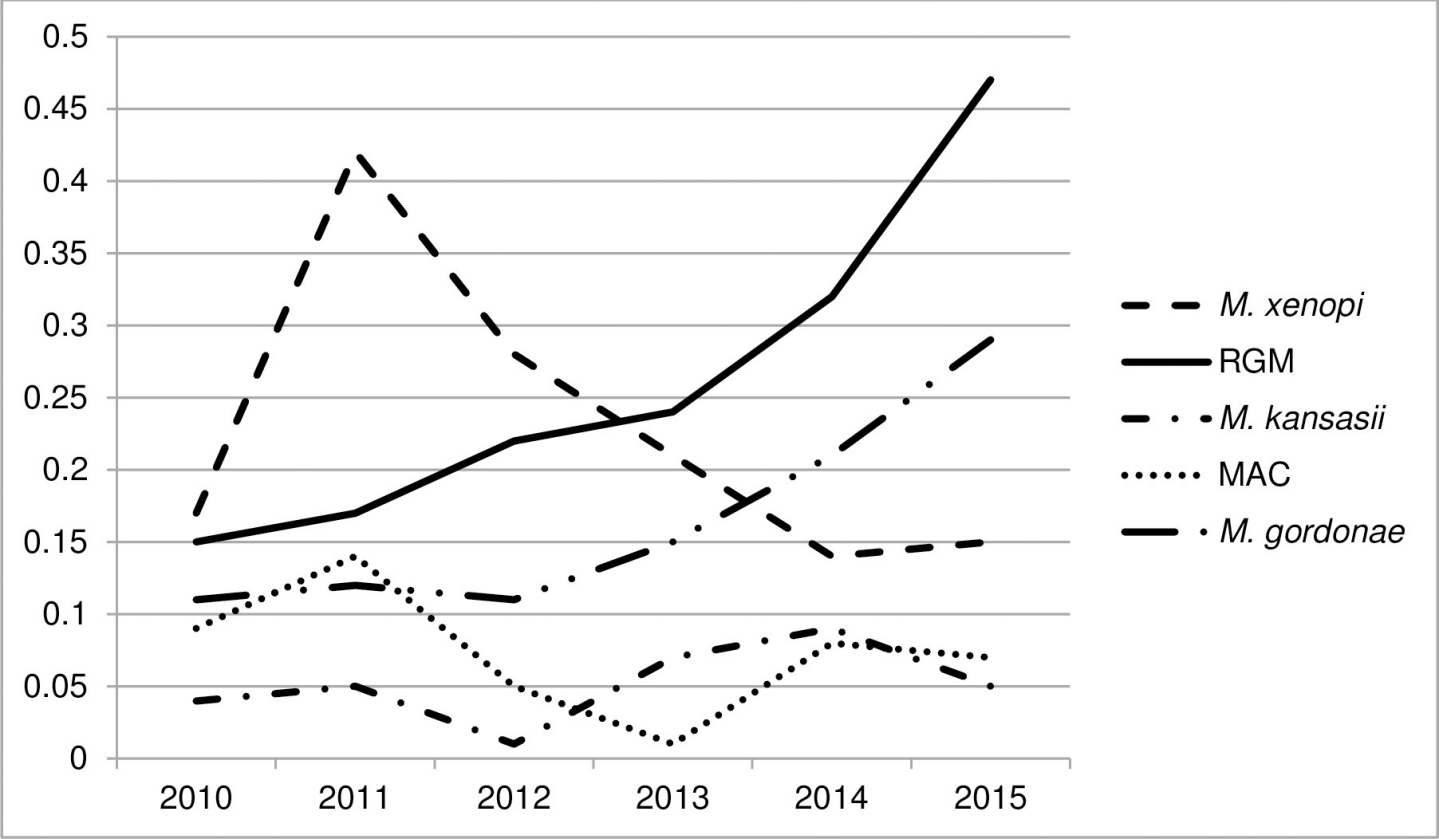
Annual isolation rates per 100,000 population of *M*. *avium* complex (MAC), *M*. *kansasii*, rapid growing mycobacteria (RGM), and *M*. *xenopi* in Serbia, 2010–2015.

Of the 565 NTM isolates, 378 identified to the species level belonged to 11 species, while the remaining 187 (33.1%) were identified as *Mycobacterium* sp. (Table 3). *M*. *xenopi* was the most frequently isolated species, followed by *M*. *gordonae*, and *M*. *fortuitum*. Although *M*. *xenopi* was the most predominant species over the study period, it showed a peak annual isolation rate in 2011 (Fig 3), but in subsequent years the rates were significantly decreasing (p = 0.0223). There has been a noticeable and constant increase in *M*. *gordonae* (p = 0.0151) and rapid growing mycobacteria (RGM) isolation rates (p = 0.0072). For all other species identified isolation rates remained without significant fluctuations over the study period, including *M*. *kansasii* and *M*. *avium* complex (MAC) rates presented in Fig 3 (p = 0.3341 and p = 0.3790, respectively).

**Table 3:** Number of nontuberculous mycobacterial (NTM) isolates stratified by species and year, Serbia, 2010–2015.

NTM species	n of incident isolates						Total n (% of 565)
	2010	2011	2012	2013	2014	2015	
*M*. *abscessus*	2	3	4	3	7	7	26 (4.6)
*M*. *chelonae*	3	6	3	3	5	3	23 (4.1)
*M*. *fortuitum*	6	3	9	11	11	24	64 (11.3)
*M*. *avium*	5	5	3	/	3	3	19 (3.4)
*M*. *intracellulare*	2	5	1	1	3	2	14 (2.5)
*M*. *gordonae*	8	9	8	11	16	21	73 (12.9)
*M*. *kansasii*	3	4	1	5	7	4	24 (4.2)
*M*. *malmoense*	/	/	/	/	1	/	1 (0.2)
*M*. *peregrinum*	1	7	8	8	7	3	34 (6.0)
*M*. *scrofulaceum*	/	2	/	/	/	/	2 (0.4)
*M*. *xenopi*	12	30	20	15	10	11	98 (17.3)
*Mycobacterium* sp.	23	40	21	29	36	38	187 (33.1)

Patient’s residence was revealed as the only demographic factor significantly correlated with distribution of distinct species (p = 0.000). Namely, *M*. *abscessus* (p = 0.007), *M*. *intracellulare* (p = 0.027), *M*. *kansasii* (p = 0.000) and *M*. *xenopi* (p = 0.000) were more prevalent in the North region, while *M*. *chelonae* (p = 0.046), *M*. *gordonae* (p = 0.030), *M*. *peregrinum* (p = 0.008), and *Mycobacterium* sp. (p = 0.028) isolates were significantly more frequently recovered from patients residing in the South region of the country (S1 Fig).

Nine different species of NTM as well as isolates identified as *Mycobacterium* sp. were recognized as causative agents of NTM PD (Table 4). *M*. *xenopi* was the most common species, and was associated with 36/126 (28.6%) cases. RGM were responsible for 35/126 (27.8%) cases, where *M*. *abscessus* contributed with 19 cases. We analyzed the overall trends of annualized rates of NTM PD cases for each species, and observed no clear trends except for increase in the rates for *M*. *kansasii* (p = 0.0097). Although the overall temporal trend for the rates of NTM PD caused by RGM was not significant (p = 0.0704), it is noteworthy that the rates considerably increased by the end of the study period.

**Table 4:** Number and incidence rates of pulmonary disease (PD) cases stratified by nontuberculous mycobacterial (NTM) species and year, Serbia, 2010–2015.

NTM species	n of PD cases (incidence rate per 100,000)						Total n (% of 126)
	2010	2011	2012	2013	2014	2015	
*M*. *abscessus*	2 (0.03)	3 (0.04)	2 (0.03)	0	7 (0.09)	5 (0.07)	19 (15.1)
*M*. *chelonae*	1 (0.01)	1 (0.01)	0	0	1 (0.01)	0	3 (2.4)
*M*. *fortuitum*	0	0	2 (0.03)	2 (0.03)	3 (0.04)	6 (0.08)	13 (10.3)
RGM	3 (0.04)	4 (0.05)	4 (0.05)	2 (0.03)	11 (0.15)	11 (0.15)	35 (27.8)
*M*. *avium*	3 (0.04)	1 (0.01)	2 (0.03)	0	2 (0.03)	2 (0.03)	10 (7.9)
*M*. *intracellulare*	2 (0.03)	1 (0.01)	0	1 (0.01)	2 (0.03)	2 (0.03)	8 (6.3)
MAC	5 (0.07)	2 (0.03)	2 (0.03)	1 (0.01)	4 (0.05)	4 (0.05)	18 (14.2)
*M*. *kansasii*	0	1 (0.01)	1 (0.01)	4 (0.05)	4 (0.05)	4 (0.05)	14 (11.1)
*M*. *malmoense*	0	0	0	0	1 (0.01)	0	1 (0.8)
*M*. *peregrinum*	0	0	1 (0.01)	2 (0.03)	1 (0.01)	0	4 (3.2)
*M*. *xenopi*	3 (0.04)	8 (0.11)	8 (0.11)	8 (0.11)	4 (0.05)	5 (0.07)	36 (28.6)
*Mycobacterium* sp.	1 (0.01)	2 (0.03)	5 (0.07)	2 (0.03)	3 (0.04)	5 (0.07)	18 (14.3)

RGM, rapid growing mycobacteria; MAC, *M*. *avium* complex.

**Fig 4:**
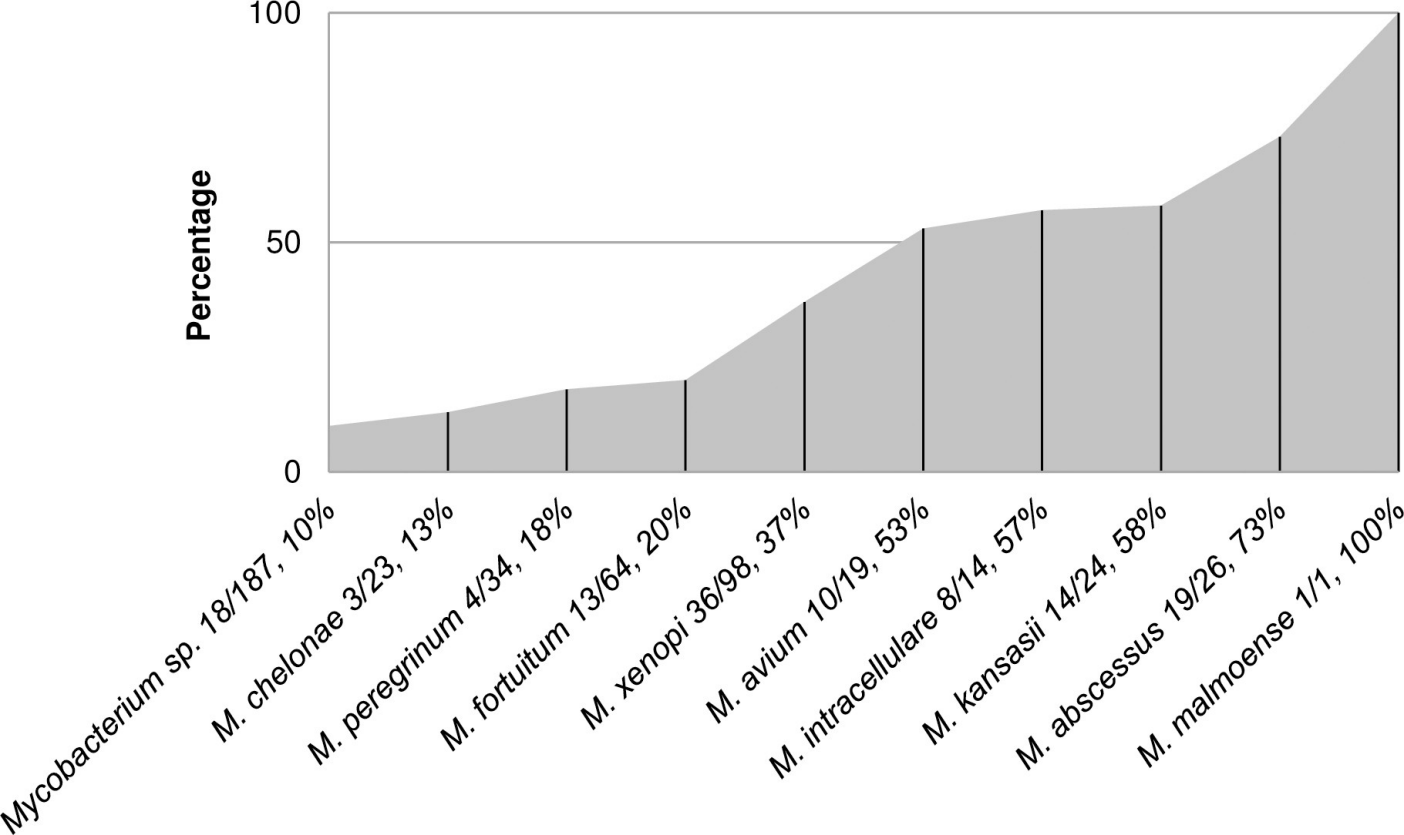
Clinical relevance of nontuberculous mycobacterial species in Serbia, 2010–2015 (number of patients who met the American Thoracic Society microbiologic diagnostic criteria/total number of patients per species, percentage of patients who met the criteria per species).

According to the proportion of patients who met the ATS/IDSA criteria out of total number of patients per species (Fig 4), the clinical relevance of 100% was established for *M*. *malmoense* only. However, we had only one case of *M*. *malmoense* isolation in our study collection, and it was clinically relevant according to the criteria we used. The species exhibiting the level of clinical relevance higher than 50% were *M*. *abscessus* (73%), *M*. *kansasii* (58%), *M*. *intracellulare* (57%), and *M*. *avium* (53%) (Fig 4). Overall, the species most likely associated with NTM PD (p = 0.000) were the aforementioned four species and, as expected, the most predominant *M*. *xenopi*.

## Discussion

The rates of NTM isolation and infection were steadily increasing over the study period in Serbia, consistent with the rising rates documented in numerous previous studies [6–8,15–21]. Annual incidence rates of NTM PD showed an unambiguously increasing trend, while the lack of the significant temporal trend in the isolation rates is obviously due to the high rate recorded in 2011. This presumably reflects immediate effects of the educational course on importance of laboratory confirmation of mycobacterial infections organized during 2010 for clinicians across the country. The proportion of clinically relevant isolates recognized according to the ATS/IDSA microbiologic criteria was 22.3%, which is similar to recently published data for Croatia, neighboring country with comparable TB burden [22]. Our average annual NTM isolation and PD rates, i.e. 1.3 per 100,000 and 0.29 per 100,000 population respectively, were lower than those reported in majority of previous studies [6,8,15,16,20–24]. The NTM PD rates we established are comparable to those reported from countries as diverse as nearby Greece [25] and Croatia [22], and faraway Brazil and New Zealand [8], which illustrates a rather variable pattern of NTM PD occurrence determined by multiple factors. We identified older age as a demographic factor strongly associated with higher NTM colonization and infection rates, which is a common finding worldwide [8,15,16,18,21,24,26]. While the rates increased significantly with age in both men and women, a significant gap between the rates among men and women was registered in the oldest age group. Predominance of men in this group is consistent with previous studies performed in European countries [7,8,16], and is most likely attributable to high prevalence of the risk factors for NTM infection such as chronic obstructive pulmonary disease (COPD) and lung neoplasm in older male population in Serbia [27]. Residence in the North region was recognized as another factor related to the higher NTM PD rates, which is concordant with more frequent occurrence of NTM species generally recognized as clinically relevant in the region.

In order to discern whether the increase in colonization and disease rates observed in the study was genuine, we analyzed data on all pulmonary samples submitted for mycobacterial culture in Serbia between 2010 and 2015. While the upward trends in NTM pulmonary isolation and infection may be partially explained by a rather steep decline in TB incidence, decreasing annual numbers of pulmonary specimens submitted for mycobacterial culture with unchanged fractions of culture-positives indicate a genuine increase in NTM isolation coincident with TB decline. This also eliminates sampling bias, i.e. increased sampling leading to increased isolation, as an indicator of changes in clinicians’ awareness of NTM relevance.

The most common NTM species isolated from pulmonary specimens were *M*. *xenopi*, *M*. *gordonae*, and *M*. *fortuitum*, which is a somewhat expected finding as these species are among the most common NTM isolated in Europe and, particularly, in neighboring Hungary, Slovenia, and Croatia [3,4,22,26]. On the other hand, MAC as the most predominant NTM both worldwide and in Europe [3,4], was relatively uncommon in Serbia. Another interesting finding is 6% of the isolates identified as *M*. *peregrinum*, which is considerably higher than the average frequency of detection of the species in European countries of up to 1% [4,16], and indicates possible presence of specific environmental reservoirs in our country. We found important difference in distribution of distinct NTM species within the country, which at least partially may be attributed to the diverse sources and routes of NTM exposure. Centralized water supply system, which has been recognized as the source of NTM species predominant in the North region such as *M*. *kansasii*, MAC and *M*. *xenopi* [9,28], is available to 82% of the region’s population (http://webrzs.stat.gov.rs). In contrast, only 57% of the South region residents have access to the public water distribution system. Exposure to NTM in soil may be presumed to be of some importance in this region where population is predominantly employed in agriculture. Our study also confirmed that isolation frequency of different species of NTM within a population is changing over time [3,10].

Five NTM species were identified as significantly associated with NTM PD. *M*. *xenopi* was the most common cause of NTM PD. It was responsible for nearly 29% of cases, which is similar to the situation in Israel [29], Ontario, Canada [6] and Croatia [22]. However, proportion of only 37% clinically relevant isolates indicates that pulmonary isolation of *M*. *xenopi* in Serbia should be interpreted with caution, i.e. should require strict adherence to preferably full ATS/IDSA criteria. Recognition of RGM as the second leading cause of NTM PD in Serbia, with 28% of all cases, is a rather unusual finding for the European region [8]. The percentage as high as the one we found is typical of Asian countries [2,8]. A particularly worrisome result is identification of *M*. *abscessus*, one of the most virulent and resistant NTM species [30], as the most clinically relevant NTM in the country. Therefore, recovery of *M*. *abscessus* from pulmonary specimens in Serbia should be considered as a high probability indicator of NTM PD. With more than 50% of clinically relevant isolates, MAC species were significantly associated with NTM PD, but the overall number of cases shows that importance of MAC in Serbia is lower than in most countries, including nearby Croatia [7,8,15,19,22]. According to the rising incidence rates and proportion of clinically relevant isolates, the NTM species clearly emerging as an important pathogen in Serbia is *M*. *kansasii*, and, thus, should be carefully monitored in the future.

The study has some limitations which have to be pointed out. First, identification of NTM colonization and infection cases was solely based on microbiologic ATS/IDSA criteria. Although this approach is considered reliable [15,19], it critically depends on laboratory methods applied. The methods used for culturing of mycobacteria in the national TB laboratory network remained primarily optimized for MTBC, and thus not preferable for recovery of numerous NTM species. It therefore seems reasonable to assume that NTM isolation frequency and consequently NTM PD rates established in our study may have been underestimated. On the other hand, misclassification of prevalent NTM PD cases as incident may have led to overestimated incidence of the disease. However, a substantial increase in NTM PD prevalence from 2011–2012 to 2014–2015 suggests that our finding of rising trend in NTM PD incidence in Serbia during the study period is valid. Second, lack of access to patients’ clinical records prevented us to evaluate co-morbidities like COPD, lung neoplasm and TB, as risk factors for NTM colonization and infection. Third, our study did not reveal the full scope of NTM species recovered from respiratory specimens, due to the national algorithm of routine laboratory diagnostics of mycobacterial infections designed to detect or rule out TB. Nearly one third of NTM isolates remained unidentified, which is considerably more than 7% of such isolates found in the European Union [4].

In conclusion, NTM PD obviously remains a rare disease in Serbia, but the rising NTM importance showed in our study justifies recognition of NTM as gradually emerging pathogens in the country. Further studies and more data are needed for more comprehensive understanding and monitoring of NTM epidemiology in the country and wider region.

## Supporting information

S1 Table Nontuberculous mycobacteria (NTM) isolation frequency rates and nontuberculous mycobacterial pulmonary disease (NTM PD) incidence rates stratified by gender and year, Serbia, 2010–2015. (DOCX)

S2 Table Nontuberculous mycobacteria (NTM) isolation frequency rates and nontuberculous mycobacterial pulmonary disease (NTM PD) incidence rates stratified by gender and age, Serbia, 2010–2015. (DOCX)

S1 Fig Regional distribution of nontuberculous mycobacterial species in Serbia, 2010–2015. (TIF)
