# Supplementary material for: Pulmonary isolation and clinical relevance of nontuberculous mycobacteria during nationwide survey in Serbia, 2010-2015
Source: PLoS One. 2018 Nov 21;13(11):e0207751. doi: 10.1371/journal.pone.0207751 (PMC6248987; doi:10.1371/journal.pone.0207751)
Supplement: S1 Table — (DOCX) [file pone.0207751.s001.docx]

**S1 Table.** **Nontuberculous mycobacteria (NTM) isolation frequency rates and nontuberculous mycobacterial pulmonary disease (NTM PD) incidence rates stratified by gender and year, Serbia, 2010-2015.**

|  | **NTM isolation frequency** | | **NTM PD incidence** | |
| --- | --- | --- | --- | --- |
| **Year** | **Male**  **n (rate)** | **Female**  **n (rate)** | **Male**  **n (rate)** | **Female**  **n (rate)** |
| 2010 | 36 (1.02) | 29 (0.78) | 5 (0.14) | 7 (0.18) |
| 2011 | 49 (1.39) | 65 (1.75) | 7 (0.20) | 10 (0.26) |
| 2012 | 41 (1.17) | 37 (1.00) | 9 (0.26) | 12 (0.32) |
| 2013 | 47 (1.35) | 39 (1.06) | 10 (0.28) | 9 (0.24) |
| 2014 | 56 (1.61) | 50 (1.37) | 17 (0.48) | 11 (0.30) |
| 2015 | 62 (1.78) | 54 (1.48) | 20 (0.57) | 9 (0.24) |
| **p value** | **0.0171** | **0.4799** | **0.0022** | **0.4756** |
