# Supplementary material for: Pulmonary isolation and clinical relevance of nontuberculous mycobacteria during nationwide survey in Serbia, 2010-2015
Source: PLoS One. 2018 Nov 21;13(11):e0207751. doi: 10.1371/journal.pone.0207751 (PMC6248987; doi:10.1371/journal.pone.0207751)
Supplement: S2 Table — (DOCX) [file pone.0207751.s002.docx]

**S2 Table. Nontuberculous mycobacteria (NTM) isolation frequency rates and nontuberculous mycobacterial pulmonary disease (NTM PD) incidence rates stratified by gender and age, Serbia, 2010-2015.**

|  | **Baseline population** | | **NTM isolation frequency** | | | **NTM PD incidence** | | |
| --- | --- | --- | --- | --- | --- | --- | --- | --- |
| **Age (years)** | **M** | **F** | **M**  **n (%)** | **F**  **n (%)** | **p value** | **M**  **n (%)** | **F**  **n (%)** | **p value** |
| 0-19 | 4401073 | 4153345 | 7 (0.0002) | 4 (0.0001) | 0.8809 | 0 (0) | 2 (0.0000) | / |
| 20-39 | 5816868 | 5615223 | 22 (0.0004) | 18 (0.0003) | 0.3669 | 5 (0.0001) | 5 (0.0001) | 0.8545 |
| 40-59 | 6001385 | 6218381 | 76 (0.0013) | 70 (0.0011) | 0.6062 | 17 (0.0003) | 20 (0.0003) | 0.6867 |
| ≥60 | 4770619 | 6127768 | 187 (0.0039) | 181 (0.0029) | 0.0046 | 45 (0.0009) | 32 (0.0005) | 0.0039 |

M, male; F, female.
