# Supplementary figures and images for: Pulmonary isolation and clinical relevance of nontuberculous mycobacteria during nationwide survey in Serbia, 2010-2015
Source: PLoS One. 2018 Nov 21;13(11):e0207751. doi: 10.1371/journal.pone.0207751 (PMC6248987; doi:10.1371/journal.pone.0207751)

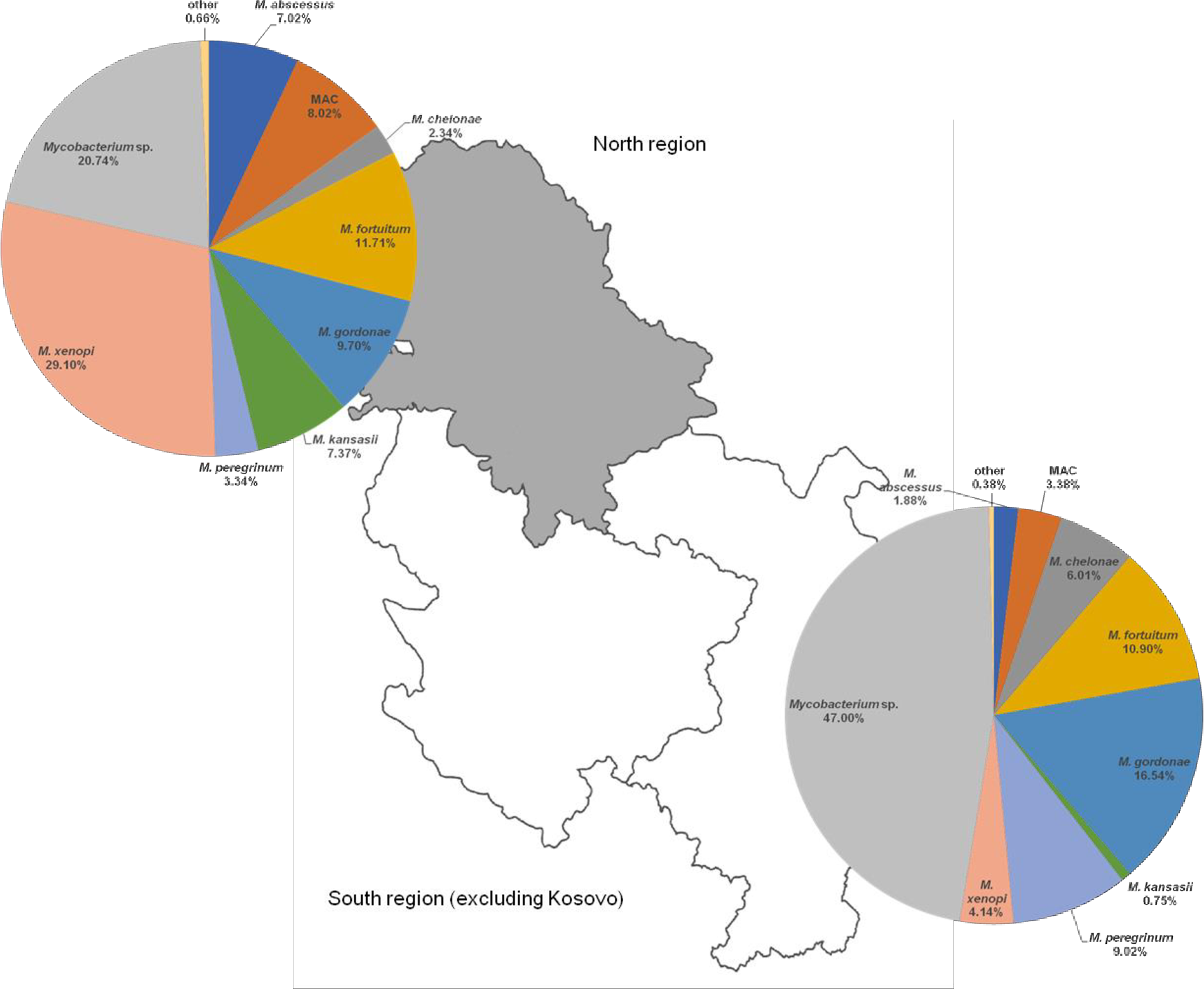

Supplement: S1 Fig — (TIF) [file pone.0207751.s003.tif]
